# Long‐Term Follow‐Up of Patients With Mitochondrial Carbonic Anhydrase VA Deficiency. A Case Report and Literature Review

**DOI:** 10.1002/jmd2.70102

**Published:** 2026-06-28

**Authors:** Shaymaa Shurrab, Amira Mobarak, Gabriella Horvath, Sylvia Stocker‐Ipsiroglu, Clara van Karnebeek, Ramona Salvarinova‐Zivkovic

**Affiliations:** ^1^ Division of Genetics and Metabolics, Department of Pediatrics McMaster University Hamilton Ontario Canada; ^2^ Division of Medical Biochemical Genetics, Department of Pediatrics Tanta University Tanta Egypt; ^3^ Division of Biochemical Genetics, Department of Pediatrics, Faculty of Medicine University of British Columbia Vancouver British Columbia Canada; ^4^ Division of Biochemical Genetics, Department of Pediatrics British Columbia Children's Hospital Vancouver British Columbia Canada; ^5^ British Columbia Children's Hospital Research Institute Vancouver British Columbia Canada; ^6^ Centre for Molecular Medicine and Therapeutics University of British Columbia Vancouver Canada; ^7^ Department of Pediatrics Amsterdam University Medical Center Amsterdam the Netherlands; ^8^ Department of Pediatrics Radboud University Medical Center Nijmegen the Netherlands; ^9^ United for Metabolic Diseases Amsterdam the Netherlands

**Keywords:** CA‐VA deficiency, encephalopathy, hyperammonemia, ketoacidosis, lactic acidosis, long‐term outcomes

## Abstract

Mitochondrial carbonic anhydrase VA (CA‐VA) deficiency is a rare inherited metabolic disorder caused by biallelic variants of the *CA5A* gene. It presents with hyperammonemia, lactic acidosis, and ketonuria, with or without hypoglycemia. We report the long‐term follow‐up of the first two reported cases of CA‐VA deficiency: a 16‐year‐old female (case 1) and her 14‐year‐old brother (case 2), both of whom presented with neonatal hyperammonemic encephalopathy, hypoglycemia, elevated lactate, ketonuria, and compensated metabolic acidosis. The diagnosis was made in both cases through the TIDEX study, which identified a homozygous pathogenic variant in the *CA5A* gene (c.697T>C, (p.Ser233Pro)). The clinical course of the index patient revealed three additional metabolic decompensation episodes (MDEs) triggered by intercurrent illnesses, with the last episode occurring at 8 years of age, highlighting the unusual timing of her MDEs compared to previously reported cases. Case 2 remained metabolically stable without any additional MDEs. These episodes were successfully treated with parenteral dextrose, a single dose of enteral carglumic acid, and occasionally parenteral lipids. Management of intercurrent illness included a sick‐day formula high in carbohydrates and fats. Currently, both cases show normal growth, development, and neurological outcomes, suggesting a favorable prognosis, and in keeping with previously reported cases. Early diagnosis of CA‐VA deficiency allows prompt treatment and prevents severe complications. With proper management, long‐term outcomes are favorable, although severe and fatal outcomes have been reported. The impact on longevity needs to be assessed over longer durations. The development of consensus management for CA‐VA deficiency is warranted.

## Key Points

- CA‐VA deficiency is a rare autosomal recessive disorder that presents with neonatal/infantile hyperammonemia, mixed acid–base disturbances, elevated lactate, and elevated ketones with or without hypoglycemia.
- There is marked intrafamilial and interfamilial variability despite identical *CA5A* genotype.
- Extended MDEs recurrence extends beyond previously reported years, indicating a need for long term follow up and management.
- Favorable long‐term neurodevelopmental and growth outcomes are achievable with early diagnosis and individualized management.
- Consensus management guidelines for CA‐VA deficiency remain an unmet need.

## 1 Introduction

**FIGURE 1:**
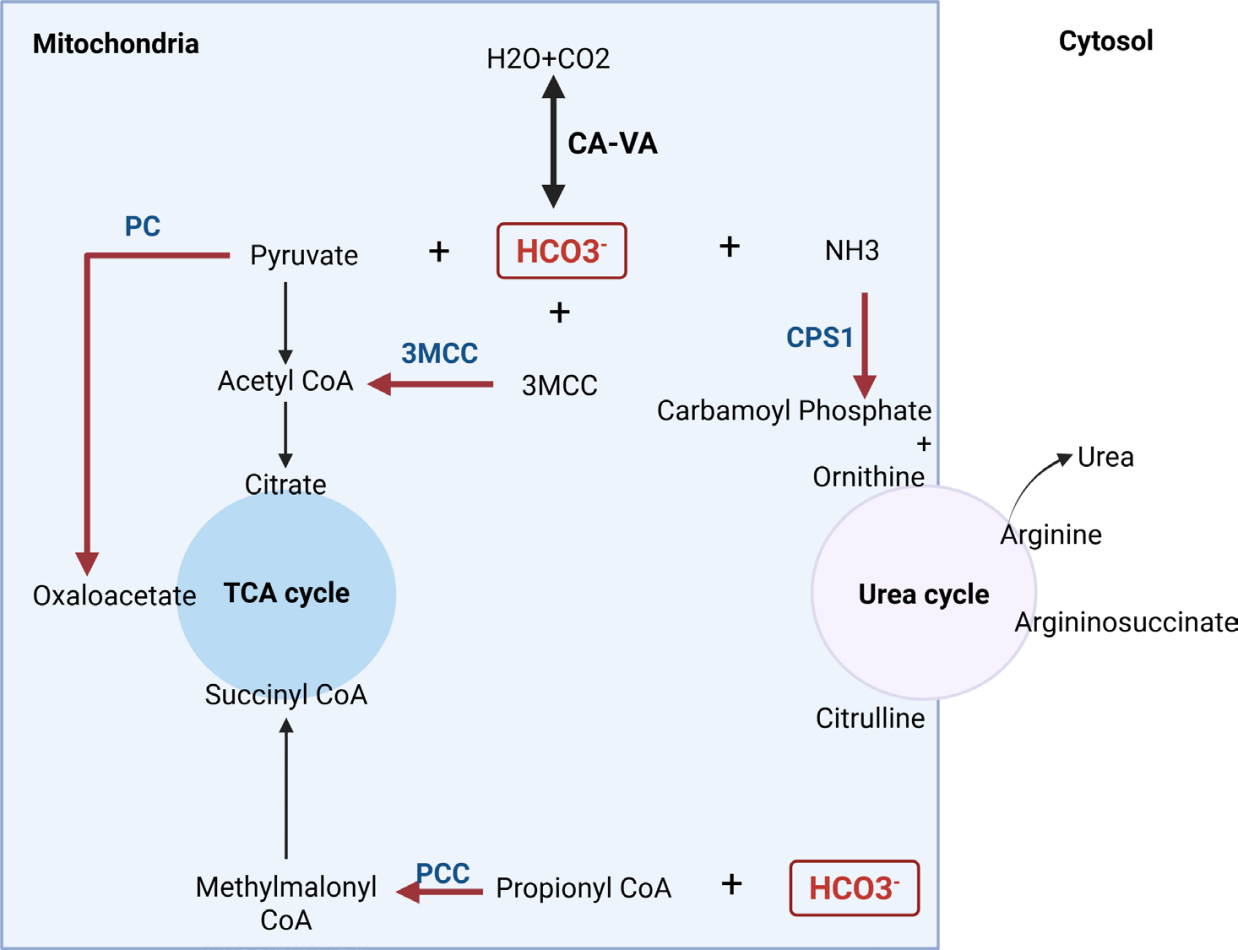
The metabolic pathways affected by CA‐VA deficiency: CA‐VA (bidirectional bolded black arrow) is responsible for the intramitochondrial production of bicarbonate (HCO_3_^−^ highlighted in red font and red‐colored boxes). There are four mitochondrial enzymes (enzymes are in blue color & reactions presented in red bolded arrows) that require HCO_3_^−^: Carbamoyl phosphate synthetase 1 (CPS1), pyruvate carboxylase (PC), propionyl CoA carboxylase (PCC), and 3‐methylcrotonyl CoA carboxylase (3MCC). Created in https://BioRender.com.

Carbonic anhydrase VA (CA‐VA) is a member of the carbonic anhydrase family. Encoded by the *CA5A* gene (OMIM # 114761) and predominantly expressed in the hepatic, renal, and skeletal muscles' mitochondria, CA‐VA catalyzes the intramitochondrial reversible hydration of carbon dioxide, producing bicarbonate (HCO_3_^−^) that is utilized in subsequent physiological processes, including ureagenesis, gluconeogenesis, and lipogenesis [1, 2]. As mitochondria are impermeable to HCO_3_^−^, its intramitochondrial production is essential for the function of 4 mitochondrial bicarbonate‐dependent enzymes (Figure 1): carbamoyl phosphate synthetase 1 (CPS1), pyruvate carboxylase (PC), propionyl CoA carboxylase (PCC), and 3‐methylcrotonyl CoA carboxylase (3MCC).

In the past decade, CA‐VA deficiency (OMIM # 615751) has been identified as a treatable cause of neonatal and infantile hyperammonemic encephalopathy. However, data on long‐term outcomes remain limited. In this report, we provide long‐term follow‐up for two previously reported cases [3, 4] and a systematic review of all 69 cases published to date.

## 2 Case Report

### 2.1 Initial Presentation

**FIGURE 2:**
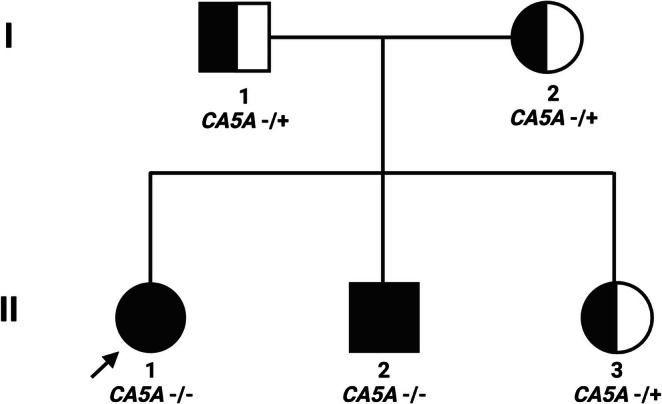
Family pedigree of cases 1 and 2: The arrow indicates the index case. The filled figures (cases II‐1 and II‐2) indicate affected individuals with CA‐VA deficiency (*CA5A* −/−, homozygous for c.697T>C). The half‐filled figures (cases I‐1, I‐2, and II‐3) indicate carriers of the *CA5A* c.697T>C (*CA5A* −/+). Created in https://BioRender.com.

Case 1 (case II‐1, Figure 2) and her brother, case 2 (case II‐2, Figure 2), were born at term following an uncomplicated pregnancy and delivery to non‐consanguineous, healthy parents of Caucasian ancestry [3]. Both cases presented on the second and first days of life, respectively, with neonatal hyperammonemic encephalopathy, hypoglycemia, elevated lactate, and a complex acid–base disturbance suggestive of metabolic acidosis with respiratory alkalosis (Table 1). Other investigations, including liver transaminases, coagulation profile, kidney function tests, and urate levels, were within the normal range.

**TABLE 1:** Summary of the investigations carried out in cases 1 and 2.

Investigations	Case 1	Case 2
Routine investigations^a^	Blood glucose: 2.2 mmol/L Sodium: 154 mmol/L (135–145) Lactate: 9.8 mmol/L (0.5–2.2) Ammonia: 438 μmol/L (< 45) Beta hydroxybutyrate: 10 μmol/L (< 0.3) Venous blood gas: pH: 7.48, pCO_2_: 10.8 mmHg, and HCO_3_^−^: 10.4 mEq/L	Blood glucose: 2.9 mmol/L Sodium: 150 mmol/L (135–145) Lactate: 8.8 mmol/L (0.5–2.2) Lactate to pyruvate ratio: 158 (10–20.7) Ammonia: 238 μmol/L (< 45) Venous blood gas: pH: 7.54, pCO_2_: 22, HCO_3_^−^: 23 mEq/L
Provincial newborn screening^a^	Negative for all screened disorders.	Negative for all screened disorders.
Plasma amino acids (μmol/L)^a^	Elevated glutamine: 1044 (243–822) Elevated proline: 475 (144–329) Elevated alanine: 811 (132–455) Low ornithine: 28 (38–207) Low citrulline: 5 (8–47) Low arginine: 17 (32–142)	Elevated glutamine 1237 (243–822) Elevated alanine: 609 (132–455) Arginine (measured following IV arginine administration): 572 (17–119) Normal citrulline: 18 (3–36) Normal ornithine: 82 (27–207)
Acylcarnitine profile (μmol/L)^a^	Unremarkable	Unremarkable
Urine organic acids (mmol/mol creatinine)^a^	Substantial elevation of lactate in urine, marked ketonuria, and mild elevation in branched‐chain keto and hydroxy acids Orotic acid was not detectable	Moderate lactic aciduria and ketonuria, significant increase in pyruvic acid, mild elevation of Krebs cycle intermediates and tyrosine metabolites, and dicarboxylic aciduria Orotic acid and uracil were within the reference range
Urine amino acids^a^	Unremarkable	Unremarkable
Biotinidase activity	Unremarkable	Unremarkable
Pyruvate carboxylase activity in fibroblasts	Not performed	Unremarkable
Respiratory chain complexes I‐V blue native electrophoresis	Not performed	Unremarkable
Brain (MRI/MRS)^a^	Unremarkable	Petechial focus of diffusion restriction adjacent to the posterior aspect of the trigone of the right lateral ventricle in keeping with a small focus of nonspecific white matter injury^b^ MRS: normal
EEG^a^	Unremarkable	Not performed
Cardiac evaluation^a^	Echocardiography: Left ventricular hypertrophy^c^	Echocardiography: Localized area of septal thickness of unknown significance^d^ ECG: Normal Sinus Rhythm
Abdominal ultrasound	No abnormalities in the liver or kidneys	No abnormalities in the liver or kidneys
Chromosomal microarray^a^	Normal female	Normal male
*NAGS* and *CPS1* gene sequencing and deletion/duplication analysis.	Unremarkable	Not performed
*TMEM70* and *PC* gene sequencing	Not performed	Unremarkable
WES	Homozygous for (NM_001739.1: c.697T>C, NP_001730.1: p.Ser233Pro) in the *CA5A* gene, ACMG classification: pathogenic	Homozygous for (NM_001739.1: c.697T>C, NP_001730.1: p.Ser233Pro) in the *CA5A* gene, ACMG classification: pathogenic

*Note:* Institution reference ranges are indicated between brackets.

Abbreviation: ACMG, American College of Medical Genetics.

^a^ Tests obtained during the initial presentation in the neonatal period.

^b^ Repeat MRI at the age of 11 years was normal.

^c^ Follow‐up echocardiography at 2, 3, 10 years of age and then annually was normal.

^d^ Findings normalized on repeat studies annually.

Following their initial presentation, both cases were transferred to a tertiary center and were admitted to the pediatric intensive care unit (PICU). Management included intravenous (IV) 10% dextrose at 150% maintenance, bicarbonate infusion to correct metabolic acidosis, and enteral carglumic acid (Carbaglu; 100 mg/kg/day) for hyperammonemia. Their symptoms resolved, and their biochemical profiles normalized upon initiation of treatment. In case 1, protein intake was restricted but increased gradually as ammonia levels normalized. Due to concerns regarding primary mitochondrial disorders, coenzyme Q10 (CoQ10; 50 mg/day), l‐carnitine (100 mg/kg/day), and vitamin C (60 mg/day) were initiated.

## 3 Results

3.1

The results of these investigations are summarized in Table 1. Both cases underwent comprehensive metabolic workups, including plasma amino acid (PAA), plasma acylcarnitine profile (ACP), urine organic acids (UOA), urine amino acids (UAA), and biotinidase activity. Additionally, PC activity in fibroblasts and respiratory chain complexes I–V blue native electrophoresis were performed in case 2. Both cases underwent brain magnetic resonance imaging and spectroscopy (MRI/MRS), echocardiography, and abdominal ultrasound, while electroencephalography (EEG) was obtained only in case 1. Whole Exome Sequencing (WES) was conducted through the Treatable Intellectual Disability Endeavor (TIDEX) study, and a homozygous variant in *CA5A* gene (NM_001739.1: c.697T>C, NP_001730.1: p.Ser233Pro) was identified and confirmed by Sanger sequencing. The variant was subsequently confirmed as pathogenic, thereby establishing the diagnosis of CA‐VA deficiency in cases 1 and 2 at ages 6 years 3 months and 4 years 3 months, respectively [3].

### 3.2 Further Metabolic Decompensation Episodes (MDEs)

Case 1 experienced three additional MDEs at ages 2 years 6 months, 3 years 6 months, and 8 years, all triggered by febrile illnesses. During these episodes, she presented with altered levels of consciousness, vomiting, hypoglycemia, massive ketonuria, elevated lactate (9.1, 8.7, and 7.3 mmol/L, respectively), hyperammonemia (peaking at 780, 420, and 266 μmol/L, respectively), and metabolic acidosis. HyperCKemia was reported during all subsequent MDEs (CK: 1414, 514, and 342 U/L, respectively; reference range: 50–305). She was treated with protein restriction, IV dextrose, IV lipids (1–2 g/kg/day), and a single dose of carglumic acid (100 mg/kg/day). Given her metabolic stability, l‐carnitine (50 mg/kg/day) was discontinued at the age of 11 years. Case 2 remained stable without additional MDEs. Ultimately, his l‐carnitine was stopped at 8 years of age.

Outside the MDEs seen in case 1, both cases experienced febrile illnesses that were successfully managed with a sick‐day formula to provide high‐calorie intake with increased lipids and carbohydrates (each 100 mL of the formula provides: 0 g of protein, 18.3 g of carbohydrate, 3.8 g of fat and 107 kcal, administered to provide 1.3 mL/kg/h and glucose infusion rate (GIR) of 4 mg/kg/min) and avoidance of fasting. Both cases continued normal/high protein intake (~4 g/kg/day), CoQ10 (150 mg/day) and vitamin C (60 mg/day) supplements, while a zinc supplement (3 mg/day) was provided intermittently during times of confirmed nutritional deficiency. The annual surveillance, including ophthalmology, cardiac, liver and renal, remained unremarkable.

### 3.3 Development, Growth, and Neurological Outcome

At age 4 years 3 months, case 1 underwent a comprehensive developmental and behavioral assessment. Her cognitive abilities, basic concept knowledge, single‐word expressive and receptive vocabulary, and visual‐motor integration were at or above average compared to her peers of the same age. Her fine motor coordination was average, with occasional fatigue‐related difficulties. However, she performed below average on the Peabody Developmental Motor Scale (Second Edition) for object manipulation, which was attributed to decreased upper‐ and lower‐body strength. Although her concurrent neurological examination did not reveal any motor weakness, she reported easy fatigability, low energy, and difficulty coping with physical activity, for which she received physiotherapy and occupational therapy. By age 5 years 4 months, her development, including motor coordination, was reported to be normal, and by age 10 years, she demonstrated significant improvement in her motor skills. At age 6 years, her speech assessment showed age‐appropriate speech and language development. Currently, she exhibits normal growth, with the last recorded measurements at age 16 years: weight of 57.9 kg (60th percentile) and height of 166.5 cm (75th percentile). Neurological examination findings were unremarkable. Although formal neurocognitive assessment was not performed, she demonstrated age‐appropriate development, academic performance, and cognitive function. Her biochemical investigations, including glucose, ammonia, lactate, ketones, CK, PAA, plasma ACP, and UOA, completely normalized outside MDEs.

Case 2, at age 4 years 5 months, underwent speech and language assessment, which revealed age‐appropriate speech, language, and fluency. His developmental assessment using the Bayley Scales of Infant and Toddler Development (third edition)—Bayley‐III indicated below‐average cognitive abilities. The Adaptive and Behavioral Assessment System (second edition)—ABAS‐II could not be completed due to increasing fatigue and distractibility, which may explain the underestimation of his cognitive abilities. Ongoing developmental monitoring revealed age‐appropriate cognitive function; thus, additional support was not indicated. At age 14 years, case 2 exhibits a normal neurological examination and an unremarkable biochemical profile. His growth was within the normal range, with the last recorded measurements of weight 67.9 kg (84th percentile) and height 180.3 cm (94th percentile). He demonstrated normal development, academic performance, and cognitive abilities (formal neurocognitive assessment was not performed).

## 4 Discussion

### 4.1 Demographic Distribution and Age of Onset

CA‐VA deficiency is a rare inherited metabolic disease caused by biallelic pathogenic variants of the *CA5A* gene. To date, 69 cases have been reported worldwide (Tables 2 and 3), including our own, spanning diverse backgrounds, with most cases originating from the Middle East and South Asia (23 cases each), suggesting founder mutations [3, 4, 5, 6, 7, 8, 9, 10, 11, 12, 15, 16, 17, 18, 19, 20, 21, 22, 23, 25, 27]. Around 81.2% of cases manifest before 2 years of age, with 66.7% presenting during the neonatal period, highlighting the impact of the physiological catabolism characteristic of this phase [3, 4, 5, 6, 7, 8, 10, 12, 13, 15, 16, 17, 18, 19, 20, 21, 22, 23, 27]. Childhood presentations account for 11.6% with onset typically ranging from 2 to 5 years [4, 11, 14, 18, 25]. While two asymptomatic adults identified through familial segregation studies have been reported [3, 18], adult‐onset presentations have not been described, indicating a predominant pediatric presentation. The remaining three cases were diagnosed antenatally [9, 24].

**TABLE 2:** Summary of CA‐VA cases reported in the literature: Focus on the genotype, molecular testing, initial presentation, newborn screening, biochemical profile, brain imaging and acute management during the initial MDE.

References	Ancestry	*CA5A* genotype	Molecular testing	Age at initial presentation	Initial presentation	Newborn screening	Biochemical profile and neuroimaging	Acute management
van Karnebeek et al. [3] and Diez‐Fernandez et al. [4]	Russian	Compound Heterozygous c.555G>A (p.Lys185Lys) & (p.His155_Leu186del) in‐frame deletion of exon 4	*CA5A* Sanger sequencing & RT‐PCR	4 DOL	Hyperammonemia Elevated lactate Hypoglycemia Metabolic acidosis Ketonuria LFTs: normal.	Not reported	ACP: normal PAA: elevated glutamine, alanine and proline. Normal citrulline, ornithine and arginine Urine orotate: normal. UOA: elevated lactate, ketones, dicarboxylic aciduria, 3‐hydroxypropionic acid, 3‐hydroxyisovaleric acid, mild elevation in 3‐methylcrotonylglycine and propionylglycine	IV lipids Protein‐free formula Carglumic acid Biotin
van Karnebeek et al. [3] and Diez‐Fernandez et al. [4]^a^	Pakistani	Homozygous 4 kb deletion encompassing exon 6 (c.619‐3427_c.774+502del4078bp, p.Asp207Gln258del)	*CA5A* Sanger sequencing	18 months	Hyperammonemia Elevated lactate Compensated metabolic acidosis	Not reported	ACP: normal PAA: normal glutamine, arginine, ornithine. Increased plasma alanine and proline Urine orotate: normal UOA: grossly increased lactate and ketones, moderately increased fumaric acid and 3‐hydroxyisovaleric acid, mildly increased 2‐a‐ketoglutaric acid	IV fluids
Diez‐Fernandez et al. [4]	Turkish	Homozygous c.123G>A (p.Trp41*)	*CA5A* & *CA5B* Sequencing	4 DOL	Hyperammonemia Elevated lactate Elevated ketones Metabolic acidosis Hypoglycemia	Not reported	ACP: not reported PAA: normal glutamine UOA: elevated lactate, ketones and pyruvate Urine orotate: normal	Carglumic acid used in parallel to other treatments
Diez‐Fernandez et al. [4]	Indian	Homozygous c.458_459+22del24bp. (p.?)	*CA5A* & *CA5B* Sequencing	5 months	Hyperammonemia Elevated lactate Elevated ketones Metabolic acidosis Normal glucose	Not reported	ACP: not reported PAA: normal glutamine UOA: massive ketonuria Urine orotate: normal	Carglumic acid not used
Diez‐Fernandez et al. [4]	Pakistani	Homozygous c.555+4_555+183del1180bp (p.?)	*CA5A* & *CA5B* Sequencing	4 years	Hyperammonemia Elevated lactate Elevated ketones Metabolic acidosis Hypoglycemia	Not reported	ACP: not reported PAA: normal glutamine UOA: massively elevated 3‐hydroxybutyric acid Urine orotate: normal	Carglumic acid not used
Diez‐Fernandez et al. [4]	Bangladeshi	Homozygous c.721G>A (p.Glu241Lys)	*CA5A* & *CA5B* Sequencing	5 DOL	Hyperammonemia Elevated lactate Elevated ketones Metabolic acidosis Normal glucose	Not reported	ACP: not reported PAA: elevated glutamine UOA: Elevated lactic acid, acetoacetic acid, 3‐hydroxy butyrate acid, 3‐hydroxyisovaleric acid and dicarboxylic aciduria Urine orotate: normal	Carglumic acid used in MDE
Diez‐Fernandez et al. [4]	Pakistani	Homozygous c.721G>A (p.Glu241Lys)	*CA5A* & *CA5B* Sequencing	4 DOL	Hyperammonemia Elevated lactate Elevated ketones Metabolic acidosis Normal glucose	Not reported	ACP: not reported PAA: elevated glutamine UOA: lactic aciduria, ketonuria, elevated pyruvate, 3‐hydroxypropionic acid and adipic acid Urine orotate: normal	Carglumic acid used in MDE Hemofiltration
Diez‐Fernandez et al. [4]	Pakistani	Homozygous c.619‐3427_c.774+502del4078bp, (p.Asp207Gln258del)	*CA5A* & *CA5B* Sequencing	2 DOL	Hyperammonemia Elevated lactate Elevated ketones Mixed metabolic acidosis & respiratory alkalosis Hypoglycemia	Not reported	ACP: not reported PAA: elevated glutamine UOA: massive excretion of lactic acid and ketones, elevated adipic acid, 3‐hydroxyisovaleric acid, slightly elevated suberic and sebacic acid Urine orotate: normal	Carglumic acid not used Hemofiltration
Diez‐Fernandez et al. [4]	Indian	Homozygous c.619‐3427_c.774+502del4078bp, (p.Asp207Gln258del)	*CA5A* & *CA5B* Sequencing	20 months	Hyperammonemia Elevated lactate Elevated ketones Glucose and blood gas not reported	Not reported	ACP: not reported PAA: glutamine 828 μM UOA: not reported Urine orotate: normal	Carglumic acid not used
Diez‐Fernandez et al. [4]	Pakistani	Homozygous c.619‐3427_c.774+502del4078bp, (p.Asp207Gln258del)	*CA5A* & *CA5B* Sequencing	3 DOL	Hyperammonemia Elevated lactate Elevated ketones Metabolic acidosis Normal glucose	Not reported	ACP: not reported PAA: elevated glutamine UOA: massive elevation in lactic acid and ketones, mild elevation in 3‐hydroxyisovaleric acid, 3‐hydroxypropionic acid, adipic acid and suberic acid Urine orotate: normal	Not reported
Diez‐Fernandez et al. [4]	Pakistani	Homozygous c.619‐3427_c.774+502del4078bp, (p.Asp207Gln258del)	*CA5A* & *CA5B* Sequencing	3 DOL	Hyperammonemia Elevated lactate Elevated ketones Metabolic acidosis Glucose not reported	Not reported	ACP: not reported PAA: elevated glutamine UOA: not reported Urine orotate: normal	Carglumic acid not used Hemofiltration
Diez‐Fernandez et al. [4]	Pakistani	Homozygous c.619‐3427_c.774+502del4078bp, (p.Asp207Gln258del)	*CA5A* & *CA5B* Sequencing	2 DOL	Hyperammonemia Elevated lactate Elevated ketones Mixed metabolic acidosis with respiratory alkalosis Normal glucose	Not reported	ACP: not reported PAA: elevated glutamine UOA: lactate and ketones grossly elevated, moderate elevation in 3‐hydroxyisovaleric acid and adipic acid, traces of propionylglycine and 3‐methylcrotonylglycine Urine orotate: normal	Carglumic acid used in parallel to other treatments Hemofiltration
Baertling et al. [5]	Not reported	Homozygous Deletion of exons 1 and 2	WES	18 months	Seizures Arterial hypertension Hypoglycemia Elevated lactate Hyperammonemia	Not reported	ACP: not reported PAA: not reported UOA: not reported Brain MRI: initially normal. Repeat: severe brain herniation MRS: lactate peak in the basal ganglia	IV Dextrose Protein and fat restriction Nitrogen scavengers (sodium benzoate and sodium phenylacetate) l‐arginine Carglumic acid
Konanki et al. [6]	Indian	Homozygous c.721G>A (p.Glu241Lys)	WES	3 DOL	Hyperammonemia Mixed metabolic acidosis and respiratory alkalosis Elevated lactate Hypoglycemia Normal urine ketones Mildly elevated LFTs	Not reported	PAA: mild elevation in tyrosine and phenylalanine ACP: normal. UOA: phenylalanine and tyrosine metabolites Brain MRI: T2 and T2 FLAIR Hyperintensities with loss of gray‐white matter differentiation. Areas of diffusion restriction involving the frontal parietal and medial occipital subcortical WM EEG: diffuse cerebral dysfunction	IV Dextrose Sodium benzoate (500 mg/kg/day) Protein restriction (1 g/kg/day)
Konanki et al. [6]^b^	Indian	Compound heterozygous c.788G>A (p.Arg263His) & c.868C>T (p.Arg290Trp).	WES	8 months	Hyperammonemia Elevated lactate Metabolic acidosis Episodes of hypoglycemia	Not reported	Not reported	IV Dextrose Sodium benzoate (250 mg/kg/day)
Konanki et al. [6]	Indian	Homozygous c.*‐56_143–1_143+1del	WES	2 DOL	Hyperammonemia Elevated lactate Hypoglycemia Metabolic acidosis No ketonuria	Not reported	ACP: normal PAA: elevated alanine and proline UOA: lactic acid, a‐2‐ketoglutaric acid, pyruvic acid, 3‐hydroxybutyric acid	Peritoneal dialysis Sodium benzoate (250 mg/kg/day) IV carnitine Oral bicarbonate
Olgac et al. [7]	Turkish	Homozygous c.721G>A (p.Glu241Lys)	WES	2 DOL	Hyperammonemia Hypoglycemia Elevated lactate HAGMA Severe ketonuria Hypernatremic dehydration Increased creatinine and BUN	Not reported	ACP: normal PAA: increased glutamine and alanine. Normal arginine, ornithine and citrulline UOA: increased lactic acid, pyruvic acid and ketone bodies. Normal 3‐hydroxypropionic acid, methyl citrate, 3‐methyl‐crotonylglycine and 3‐hydroxyisovaleric acid	IV Dextrose (GIR 10 mg/kg/min) Sodium benzoate (250 mg/kg/day) Bicarbonate infusion l‐carnitine Biotin Hydroxocobalamin Carglumic acid Hemofiltration
Marwaha et al. [8]	Sri‐Lankan	Homozygous c.721G>A (p.Glu241Lys)	*CA5A* targeted gene sequencing	3 DOL	Hyperammonemia Elevated lactate Massive ketonuria HAGMA	Normal	ACP: low free and total carnitine PAA: elevated alanine. Normal arginine and citrulline UOA: lactic and ketoaciduria, dicarboxylic aciduria, liver metabolites Brain MRI: laminar subdural hematomas in the parietooccipital regions, posterior fossa, and posterior falx. Nonspecific hyperintensities in the deep WM in frontal, parietal and occipital lobes	IV Dextrose Bicarbonate infusion Nitrogen scavengers (sodium benzoate and sodium phenylacetate) l‐arginine l‐carnitine Carglumic acid
Marwaha et al. [8] and Ibrahim et al. [9]	Indian	Compound heterozygous c.721G>A (p.Glu241Lys) & c.619‐?_774+?del	Hyperammonemia gene panel Confirmed by Sanger sequencing and qPCR	2 DOL	Hyperammonemia Hypoglycemia Mixed metabolic acidosis and respiratory alkalosis Elevated lactate Elevated ketones	Normal	ACP: low free carnitine, elevated C4‐OH and C6‐OH PAA: elevated alanine and glutamine. Low arginine. Normal citrulline UOA: massive lactic aciduria, ketonuria, dicarboxylic aciduria, 3‐hydroxy‐3‐methylsuccinic aciduria, pyruvic aciduria, 3‐hydroxyisovaleric acid. Mild elevation in 3‐methylcrotonylglycine, tiglylglycine, and 2‐ethyl‐3‐hydroxypropionic acid Orotic acid slightly elevated Brain MRI (4 DOL): normal	IV Dextrose IV lipids Nitrogen scavengers (sodium benzoate and sodium phenylacetate) l‐arginine Carglumic acid (100 mg) Biotin (5 mg) Hemofiltration
Miller et al. [10]^c^	Not reported	Compound heterozygous Out‐of‐frame deletion spanning exon 3 & c.473A>C p.(His158Pro)	Hyperammonemia gene panel Deletion/duplication analysis of *CA5A*	4 DOL	Hyperammonemia Lactic acidosis	Not reported	Not reported	Low protein feeds l‐carnitine
Miller et al. [10]^c^	Not reported	Compound heterozygous Out‐of‐frame deletion spanning exon 3 & c.473A>C p.(His158Pro).	Hyperammonemia gene panel Deletion/duplication analysis of *CA5A*	19 months	Hyperammonemia Elevated lactate Hypoglycemia Ketonuria Metabolic acidosis	Normal	ACP: not reported PAA: low arginine, citrulline and ornithine with high alanine and proline, normal glutamine UOA: increased lactate, ketones, 3‐hydroxypropionic acid and 3‐methylcrotonylglycine Brain MRI: T2 FLAIR signal abnormalities in the bifrontal cortex and bilateral medial occipital cortex MRS: elevated glutamate and lactate	IV Dextrose (8 mg/kg/min) IV insulin (0.05 unit/kg/h) Nitrogen scavengers (sodium benzoate and sodium phenylacetate 250 mg/kg/day) l‐arginine HCl (200 mg/kg/day)
Sredkova et al. [11] (Poster Presentation ICIEM 2021)	Not reported	Compound heterozygous c.555G>A (p.Lys185 =) & deletion in exon 1	WES followed by *CA5A* gene analysis for deletion and duplication assessment	3‐years‐7‐months	Hyperammonemia Hyperglycemia Elevated lactate Metabolic acidosis switched to respiratory alkalosis Normal liver function tests	Not reported	ACP: not reported PAA: not reported UOA: elevated lactate, ketone bodies and 3‐methylcrotonylglycinuria	IV Dextrose Sodium phenylbutyrate l‐arginine HCl l‐carnitine Carglumic acid
Singanamalla et al. [12]	Indian	Compound heterozygous c.123G>T (p.Trp41Cys) & c.690C>T (p.Tyr230 =)	WES Confirmed by Sanger sequencing	3 DOL	Cardiac arrest and cyanosis Hyperammonemia Elevated lactate Metabolic acidosis Ketonuria Normal glucose	Not reported	ACP: normal PAA: normal UOA: normal Brain MRI: normal	IV Dextrose Sodium benzoate Carnitine Biotin Riboflavin Folic acid Zinc
Sequeira [13]	Caucasian	Compound heterozygous c.774G>A (p.Gln258His) & c.(142+1_143–1)_(459+1_460–1)del (p.?)	*CA5A* gene sequencing with MLPA	2 DOL	Hyperammonemia Metabolic acidosis Elevated lactate Hypoglycemia Elevated ketones	Normal	ACP: not reported PAA: elevated glutamine and alanine, low/normal citrulline. UOA: normal	IV Dextrose Protein‐free formula Nitrogen scavengers (sodium benzoate and sodium phenylacetate) l‐arginine Carglumic acid (used with rebound hypoglycemia)
Stockdale et al. [14]	Not reported	Homozygous c.619_3420_c.774+502del4078pb (p.Asp207_Gln258del)	*GA5A* targeted gene sequencing	2‐years‐8‐months	Hyperammonemia Elevated lactate Ketonuria	Not reported	ACP: evidence of ketogenesis PAA: normal glutamine and alanine on initial presentation Urine orotic acid: normal UOA: ketonuria Brain MRI: bilateral cerebral cortical cytotoxic edema	Nitrogen scavengers Carglumic acid (single dose)
Ibrahim et al. [9]	Indian	Compound heterozygous c.721G>A (p.Glu241Lys) c.619‐?_774+?del	FVT Diagnosed antenatally	N/A	N/A	Not reported	ACP: not reported PAA: not reported UOA: normal	IV Dextrose IV lipids (2 g/kg/day) Carglumic acid (100 mg/kg twice daily)
Semenova et al. [15]	Russian	Homozygous c.555G>A, p.Lys185Lys	WGS Confirmed by Sanger sequencing	3 DOL	Seizures Intestinal bleeding Significant liver disease Hypoglycemia Elevated lactate HAGMA Elevated ketones Elevated LFTs (×10 folds above normal)	Not reported	ACP: normal PAA: Elevated proline and alanine, normal citrulline, ornithine and arginine UOA: Mild elevation in pyruvate in urine, marked elevation in lactate, 3‐hydroxybutyric acid and 2‐ketoglutaric acid EEG: Diffuse cerebral dysfunction, electrographic generalized seizures	IV fluids Bicarbonate l‐carnitine Cytoflavin (succinic acid, nicotinamide, inosine & riboflavin)
Urmila et al. [16]^d^	Indian	Homozygous c.59G>A (p.Trp20*).	WES	3 DOL	Refractory seizures (tonic & multi‐focal clonic seizures) Neonatal Hypoglycemia Elevated lactate Hyperammonemia	Not reported	ACP: normal PAA: not reported UOA: normal Brain MRI: mild cortical atrophy EEG: not available	IV Dextrose Multiple AEMs Inotropes for poor perfusion
Ganesh and Narayanan [17]^d^	Indian	Homozygous Missense mutation in exon 6	Not reported	3 months	Generalized seizures Moderate hepatomegaly Hyperammonemia Metabolic acidosis Elevated lactate Elevated LFTs	Not reported	ACP: normal PAA: normal UOA: normal. Brain MRI: normal	IV fluids Sodium benzoate Carnitine Bicarbonate Peritoneal dialysis
Al‐Thihli et al. [18]^e^	Omani	Homozygous c.59G>A (p.Trp20*)	WES	1 DOL	Hyperammonemia Elevated lactate Normal ketones Normal blood glucose Elevated CK	Not reported	ACP: not reported PAA: significantly elevated glutamine, proline, alanine and mildly elevated methionine UOA: lactic aciduria, ketonuria and mild elevation in 3‐hydroxyisovaleric acid Brain MRI: brain edema with focal areas of diffusion restriction in the right lentiform nucleus and the right thalamus in keeping with acute infarcts	Ammonia scavenger (sodium benzoate & sodium phenylbutyrate) Carglumic acid (ammonia normalized within 24 h when used alone)
Al‐Thihli et al. [18]^e^	Omani	Homozygous c.59G>A (p.Trp20*)	WES	4 DOL	Hyperammonemia Elevated lactate Hypoglycemia Normal ketones Elevated CK	Not reported	ACP: not reported PAA: Isolated elevated ornithine UOA: lactic aciduria, ketonuria, 3‐hydroxyisovaleric aciduria Brain MRI (9 DOL): abnormal T2 signal intensity and diffusion restriction in the periorlandic cortex, insula, and basal ganglia in keeping with hyperammonemic encephalopathy	Ammonia scavenger (sodium benzoate & sodium phenylbutyrate) Carglumic acid (ammonia normalized within 24 h when used alone)
Al‐Thihli et al. [18]^e^, ^f^	Omani	Homozygous c.59G>A (p.Trp20*)	GA5A targeted gene sequencing	3 DOL	Hyperammonemia Elevated lactate Hypoglycemia Normal ketones Elevated CK	Not reported	ACP: not reported PAA: normal UOA: normal	Ammonia scavenger (sodium benzoate & sodium phenylbutyrate) Carglumic acid (ammonia normalized within 24 h when used alone)
Al‐Thihli et al. [18]^e^, ^f^	Omani	Homozygous c.59G>A (p.Trp20*)	GA5A targeted gene sequencing	2 DOL	Hyperammonemia Elevated lactate Normal ketones	Not reported	ACP: not reported PAA: normal UOA: not reported Brain MRI: Changes consistent with hyperammonemic encephalopathy	Ammonia scavenger (sodium benzoate & sodium phenylbutyrate) Carglumic acid (ammonia normalized within 24 h when used alone)
Al‐Thihli et al. [18]^e^	Omani	Homozygous c.59G>A (p.Trp20*)	GA5A targeted gene sequencing	3 DOL	Hyperammonemia Elevated lactate Normal ketones Elevated CK	Not reported	ACP: not reported PAA: normal UOA: not reported Brain CT: normal	Ammonia scavenger (sodium benzoate & sodium phenylbutyrate) Carglumic acid (ammonia normalized within 24 h when used alone)
Al‐Thihli et al. [18]^e^	Omani	Homozygous c.59G>A (p.Trp20*)	GA5A targeted gene sequencing	3 DOL	Hyperammonemia Elevated lactate Normal ketones	Not reported	ACP: not reported PAA: normal UOA: not reported Brain CT: normal	Ammonia scavenger (sodium benzoate & sodium phenylbutyrate) Carglumic acid (ammonia normalized within 24 h when used alone)
Al‐Thihli et al. [18]^e^	Omani	Homozygous c.59G>A (p.Trp20*)	GA5A targeted gene sequencing	2 DOL	Hyperammonemia Blood glucose, ketones and lactate were not reported	Not reported	ACP: not reported PAA: normal UOA: not reported Brain CT: normal	Ammonia scavenger (sodium benzoate & sodium phenylbutyrate) Carglumic acid (ammonia normalized within 24 h when used alone)
Al‐Thihli et al. [18]^e^	Omani	Homozygous c.59G>A (p.Trp20*)	GA5A targeted gene sequencing	3 DOL	Hyperammonemia Blood glucose, ketones and lactate were not reported	Not reported	ACP: not reported PAA: normal UOA: not reported	Ammonia scavenger (sodium benzoate & sodium phenylbutyrate) Carglumic acid (ammonia normalized within 24 h when used alone)
Al‐Thihli et al. [18]^e^	Omani	Homozygous c.59G>A (p.Trp20*)	GA5A targeted gene sequencing	5 years	Hyperammonemia Elevated lactate Hypoglycemia Normal ketones	Not reported	ACP: not reported PAA: not reported UOA: not reported	Ammonia scavenger (sodium benzoate & sodium phenylbutyrate) Carglumic acid (ammonia normalized within 24 h when used alone)
Al‐Thihli et al. [18]^e^	Omani	Homozygous c.59G>A (p.Trp20*)	WES	2 DOL	Hyperammonemia Elevated lactate Elevated CK Normal glucose Normal ketones	Not reported	ACP: not reported PAA: not reported UOA: not reported	Ammonia scavenger (sodium benzoate & sodium phenylbutyrate) Carglumic acid (ammonia normalized within 24 h when used alone)
Al‐Thihli et al. [18]^e^	Omani	Homozygous c.59G>A (p.Trp20*)	GA5A targeted gene sequencing	3 DOL	Hyperammonemia Elevated lactate Elevated ketones Elevated CK Normal glucose	Not reported	ACP: not reported PAA: not reported UOA: not reported	Ammonia scavenger (sodium benzoate & sodium phenylbutyrate) Carglumic acid (ammonia normalized within 24 h when used alone)
Al‐Thihli et al. [18]	Omani	Homozygous c.59G>A (p.Trp20*)	GA5A targeted gene sequencing	3 DOL	Elevated CK Ammonia, lactate, glucose and ketones are not reported	Not reported	ACP: not reported PAA: not reported UOA: not reported	Not reported
Al‐Thihli et al. [18]	Omani	Homozygous c.59G>A (p.Trp20*)	GA5A targeted gene sequencing	3 DOL	Elevated CK Ammonia, lactate, glucose and ketones are not reported	Not reported	ACP: not reported PAA: not reported UOA: not reported	Not reported
Al‐Thihli et al. [18]^e^	Omani	Homozygous c.59G>A (p.Trp20*)	GA5A targeted gene sequencing	3 DOL	Hyperammonemia CK, lactate, glucose and ketones are not reported	Not reported	ACP: not reported PAA: not reported UOA: not reported	Ammonia scavenger (sodium benzoate & sodium phenylbutyrate) Carglumic acid (ammonia normalized within 24 h when used alone)
Al‐Thihli et al. [18]^g^	Omani	Homozygous c.59G>A (p.Trp20*)	GA5A targeted gene sequencing	N/A	Ammonia normal Lactate, glucose, CK and ketones are not reported	Not reported	N/A	N/A
Al‐Thihli et al. [18]^g^	Omani	Homozygous c.59G>A (p.Trp20*)	GA5A targeted gene sequencing	N/A	Ammonia normal Lactate, glucose, CK and ketones are not reported	Not reported	N/A	N/A
Al‐Thihli et al. [18]^e^	Omani	Homozygous c.59G>A (p.Trp20*)	GA5A targeted gene sequencing	4 years	Hyperammonemia Elevated lactate Elevated ketones Normal glucose Normal CK	Not reported	ACP: not reported PAA: not reported UOA: not reported	Ammonia scavenger (sodium benzoate & sodium phenylbutyrate) Carglumic acid (ammonia normalized within 24 h when used alone)
Al‐Thihli et al. [18]^e^	Omani	Homozygous c.59G>A (p.Trp20*)	WES	18 months	Hyperammonemia Elevated lactate Hypoglycemia Elevated ketones Elevated CK	Not reported	ACP: not reported PAA: not reported UOA: not reported	Ammonia scavenger (sodium benzoate & sodium phenylbutyrate) Carglumic acid (ammonia normalized within 24 h when used alone)
Mathew et al. [19]	Indian	Compound heterozygous c.721G>A (p.Glu241Lys) & c.619‐3420_c.774+502del4078bp spanning introns 5 & 6 and exon 6	WES followed by sanger sequencing for *CA5A* qPCR and LR‐PCR	3 DOL	Multifocal clonic seizures progressed into status epilepticus Metabolic acidosis with respiratory alkalosis Hyperammonemia Elevated lactate Elevated liver transaminases (mild) Normal ketones Normal glucose Normal CK	Not reported	ACP: not reported PAA: normal UOA: normal Brain MRI: WM hyperintensities in globus pallidus. MRS: choline peak	IV fluids Inotropes (dopamine and adrenaline) AEM
Phadke et al. [20]	Indian	Homozygous c.721G>A (p.Glu241Lys)	WES	4 DOL	Hyperammonemia Hypoglycemia Elevated lactate Metabolic acidosis	Not reported	ACP: non diagnostic PAA: non diagnostic UOA: non diagnostic	IV Dextrose Nitrogen scavengers (sodium benzoate) Peritoneal dialysis
Majety et al. [21]	Indian	Homozygous c.721G>A (p.Glu241Lys)	WES	1 DOL	Severe persistent metabolic acidosis hypoammonemia Elevated lactate Elevated ketones Normal glucose Normal LFTs	Not reported	ACP: Normal total carnitine with low free carnitine, normal acylcarnitine levels PAA: Elevated alanine, tyrosine, methionine and phenylalanine UOA: Elevated urine lactic acid, pyruvic acid and ketones	IV fluids Bicarbonate infusion Inotropes Hydrocortisone Inhaled nitric oxide
Abdulwahhab et al. [22]	Indian	Homozygous Deletion of exon 6	WES	3 DOL	Hyperammonemia Hypoglycemia Elevated lactate Elevated ketones HAGMA	Not reported	ACP: mild nonspecific elevations, normal carnitine levels PAA: Elevated alanine, glutamine and proline without accumulation of urea cycle products Urine orotic acid: normal UOA: Elevated pyruvate and pyroglutamic acid, elevated lactate and ketone bodies.	IV Dextrose IV ammonia scavengers (sodium Benzoate and sodium phenylacetate) Carglumic acid (100 mg/kg/day) Biotin Riboflavin CoQ10 Feeding: EBM Hemofiltration for 24 h
Keehan et al. [23]	Guatemalan	Homozygous c.475T>C (p.Trp159Arg)	WES	18 h	Hyperammonemia Hypoglycemia HAGMA compensated with respiratory alkalosis Lactic acidosis Elevated ketones	Normal	PAA: elevated glutamine, glycine, alanine, proline, and asparagine. ACP: low free carnitine, elevated acetylcarnitine, non‐specific elevation in medium and long chain acylcarnitines secondary to mitochondrial dysfunction UOA: marked elevation in lactic acid and ketones	IV Dextrose IV nitrogen scavengers (sodium Benzoate and sodium phenylacetate) IV l‐arginine HCl. l‐carnitine Cyanocobalamin Riboflavin Biotin Thiamine
Manoy et al. [24]	North American/Russian	Compound Heterozygous c.198_208delins ACCCGG (p.Ile67Profs*13) & c.454G>A (p.Ala142Thr)	Gene panel	4 DOL	Normal glucose HAGMA Hyperammonemia Lactic acidosis Elevated ketones Mildly elevated LFTs	Normal	PAA: Elevated glutamine and alanine. Normal citrulline and lysine. ACP: elevated 3‐hydroxybutyrylcarnitine, normal propionylcarnitine and isovalerylcarnitine UOA: elevated lactate, ketones, 2‐ketoisovalerate and 2‐ketoisocaproate	IV glucose IV lipids Nitrogen scavengers Bicarbonate infusion Cofactor supplementation Carnitine supplementation Hemofiltration
Manoy et al. [24]	North American/Russian	Compound Heterozygous c.198_208delins ACCCGG (p.Ile67Profs*13) & c.454G>A (p.Ala142Thr)	FVT Diagnosed antenatally	N/A	Normal serial ammonia and blood gas	Normal	Not reported	Delivery at tertiary hospital Strict breast feeding with supplementary formula/EBM Carglumic acid 100 mg/kg/day for 5 days
Manoy et al. [24]	North American/Russian	Compound Heterozygous c.198_208delins ACCCGG (p.Ile67Profs*13) & c.454G>A (p.Ala142Thr)	FVT Diagnosed antenatally	N/A	Mild respiratory distress Normal serial ammonia and blood gas	Normal	Not reported	Delivery at tertiary hospital Strict breast feeding with supplementary formula/EBM. Carglumic acid 100 mg/kg/day for 5 days
Bin Hadyan et al. [25]	Saudi	Homozygous Deletion involving exons 3–7	WES	3 years	Global developmental delay Axial hypotonia No history of MDEs Mildly elevated lactate Normal ammonia	Not reported	Normal biochemical profile including PAA, ACP, UOA	N/A
Bin Hadyan et al. [25]^h^	Saudi	Homozygous Deletion involving exons 3–7	WGS	3 years	Developmental delay affecting language and motor milestones No history of MDEs Normal ammonia, lactate, CK, LFTs and KFTs	Not reported	Normal biochemical profile including PAA, ACP, UOA Brain MRI: marked thinning of corpus callosum, mild periventricular leukomalacia	N/A
Bin Hadyan et al. [25]^i^	Saudi	Homozygous Deletion involving exons 3–7	WGS	4 years	Mild speech delay No history of MDEs Normal ammonia, lactate, CK, LFTs and KFTs	Not reported	Normal biochemical profile including PAA, ACP, UOA Brain MRI: minor abnormalities	N/A
Fragoso et al. [26]	Not reported	Compound heterozygous c.472C>T (p.His158Tyr) & c.556C>T (p.Leu186Phe)	Not reported	2 DOL	Hyperammonemia Elevated lactate Normal glucose	Not reported	ACP: not reported PAA: elevated glutamine UOA: increased excretion of lactic acid, pyruvic acid and metabolites associated with ketosis Urine orotic acid: not detected Brain MRI: pattern consistent with hyperammonemia (selective involvement of cortical and juxtacortical regions of the insular/peri‐nsular area, Sylvian/peri‐Sylvian fissures, and the peri‐orlandic cortex)	Not reported
Fragoso et al. [26]	Not reported	Homozygous Deletion of exon 1	Not reported	3 DOL	Hyperammonemia Elevated lactate Hypoglycemia	Not reported	ACP: not reported PAA: elevated glutamine UOA: increased excretion of lactic acid, pyruvic acid and metabolites associated with ketosis Urine orotic acid: not detected Brain MRI: pattern consistent with hyperammonemia (selective involvement of cortical and juxtacortical regions of the insular/peri‐nsular area, Sylvian/peri‐Sylvian fissures, and the peri‐orlandic cortex)	Not reported
Fragoso et al. [26]^j^	Not reported	Homozygous c.742C>T	Not reported	3 months	Severe tonic clonic seizures Hyperammonemia Elevated lactate Compensating metabolic acidosis Normal glucose	Not reported	ACP: not reported PAA: elevated glutamine UOA: increased excretion of lactic acid and metabolites associated with ketosis Urine orotic acid: not detected Brain MRI: extensive involvement affecting the basal ganglia and the brain stem	Not reported
Fragoso et al. [26]	Not reported	Homozygous Single exon deletion	Not reported	13 months	Hyperammonemia Normal glucose	Not reported	ACP: not reported PAA: not reported UOA: increased metabolites associated with ketosis and other organic acids (secondary disturbances) Urine orotic acid: not detected Brain MRI: pattern consistent with hyperammonemia (selective involvement of cortical and juxtacortical regions of the insular/peri‐nsular area, Sylvian/peri‐Sylvian fissures, and the peri‐orlandic cortex)	Not reported
Fragoso et al. [26]	Not reported	Homozygous c.59G>A (p.Trp20*)	Not reported	1 DOL	Severe tonic clonic seizures Hyperammonemia Elevated lactate Normal glucose	Not reported	ACP: not reported PAA: not reported UOA: increased excretion of lactic acid, pyroglutamate, phenylacetic acid, 4‐OH‐phenyllactic acid, and 4‐OH‐phenylpyruvic acid Urine orotic acid: not detected Brain MRI: extensive involvement affecting the basal ganglia and the brain stem	Not reported
Fragoso et al. [26]	Not reported	Homozygous c.59G>A (p.Trp20*)	Not reported	4 DOL	Hyperammonemia Elevated lactate Hypoglycemia Metabolic acidosis	Not reported	ACP: not reported PAA: normal UOA: not reported Brain MRI: involvement of cortical and juxtacortical regions of the insular/peri‐nsular area, Sylvian/peri‐Sylvian fissures, and the peri‐orlandic cortex, extensive involvement affecting the basal ganglia and the brain stem	Not reported
Fragoso et al. [26]	Not reported	Homozygous c.59G>A (p.Trp20*)	Not reported	3 DOL	Hyperammonemia Normal glucose	Not reported	ACP: not reported PAA: not reported UOA: not reported Brain MRI (2nd MDE): selective bilateral restriction diffusion in the claustrum.	Not reported
Fragoso et al. [26]	Not reported	Homozygous c.59G>A (p.Trp20*)	Not reported	6 DOL	Hyperammonemia Elevated lactate Normal glucose Metabolic acidosis	Not reported	ACP: not reported PAA: not reported UOA: not reported	Not reported
Fragoso et al. [26]^f^, ^k^	Not reported	Homozygous c.59G>A (p.Trp20*)	Not reported	3 DOL	Hyperammonemia Elevated lactate Severe recurrent hypoglycemia	Not reported	ACP: not reported PAA: not reported UOA: not reported Brain MRI: involvement of cortical and juxtacortical regions of the insular/peri‐nsular area, Sylvian/peri‐Sylvian fissures, and the peri‐orlandic cortex, extensive involvement affecting the basal ganglia and the brain stem	Not reported

Abbreviations: ACP, acylcarnitine profile; BUN, blood urea nitrogen; BW, birth weight; CK, creatinine kinase; EBM, expressed breast milk; FVT, familial variant testing; HAGMA, high anion gap metabolic acidosis; KFTs, kidney function tests; LFTs, liver function tests; LR‐PCR, long‐range polymerase chain reaction; MDEs, metabolic decompensation episodes; MLPA, multiplex ligation‐dependent probe amplification; N/A, not applicable; OA, organic acidemias; qPCR, quantitative polymerase chain reaction; UCD, urea cycle defects; UOA, urine organic acids; WES, whole exome sequencing; WGS, whole genome sequencing; WM, white matter.

^a^ Patient's older brother (17 years) was homozygous for the same variant and declined further follow up. Prior MDEs cannot be excluded given the limited history.

^b^ Muscle biopsy: complex 1 deficiency.

^c^ Initial hyperammonemia gene panel missed the deletion in the second allele.

^d^ Unclear whether these investigations were obtained during the MDE or after treatment.

^e^ The paper did not specify treatment modalities for each patient.

^f^ Patients have a concurrent diagnosis of medium chain acyl‐coenzyme A dehydrogenase (MCAD) deficiency.

^g^ Asymptomatic adults diagnosed via cascade family testing.

^h^ Patient also had two other likely pathogenic variants: *SYT2* (homozygous) causes AD/AR congenital myasthenia syndrome 7A/7B and *SYNE4* (heterozygous) causes AR deafness. No clinical correlation was found.

^i^ Patient also had two other likely pathogenic variants: *SYT2* (homozygous) AD/AR congenital myasthenia syndrome7A/7B & *SYNE4* (homozygous) causes AR deafness. No clinical correlation was found.

^j^ Patient has *CPS1* variant (c.2549C>T, p.Arg850Leu)—not diagnostic.

^k^ Patient is homozygous for *ACADM* variant (c.449_452del, p.Thr150Argfs*).

**TABLE 3:** Summary of CA‐VA cases reported in the literature: Additional MDEs, long‐term follow‐up, neurological outcome and long‐term management.

References	Number of additional MDEs	Age of last reported MDE	Age at the time of publication	Total number of MDEs	Long‐term follow‐up and neurological outcome	Long‐term management	
						Dietary management	Medications
van Karnebeek et al. [3] and Diez‐Fernandez et al. [4]	None	4 DOL	6 months	1	Normal growth and development	On breast feeding, no protein restriction Sick‐day plan	Continued Carglumic acid until 4 months, then discontinued
van Karnebeek et al. [3] and Diez‐Fernandez et al. [4]^a^	1	16 months	11 years	2	Minor LD (no formal testing) Normal growth and good developmental progress	Discharged on protein restriction after second MDE	Started on sodium benzoate and l‐arginine after the first MDE (at 16 months of age), treatment withdrawn at age 7 years
Diez‐Fernandez et al. [4]	None	4 DOL	12 months	1	Normal	Not reported	Not reported
Diez‐Fernandez et al. [4]^b^	1	2 years	10 years	2	Normal	Not reported	Not reported
Diez‐Fernandez et al. [4]^b^	1	4‐years‐1‐month	10 years	2	Normal	Not reported	Not reported
Diez‐Fernandez et al. [4]	None	5 DOL	Not reported	1	Normal	Not reported	Not reported
Diez‐Fernandez et al. [4]	None	4 DOL	3 years	1	Normal	None	None
Diez‐Fernandez et al. [4]^b^	1	23 months	6 years	2	Normal	Sick‐day plan	None
Diez‐Fernandez et al. [4]	None	20 months	4 years	1	Normal	Not reported	Not reported
Diez‐Fernandez et al. [4]	None	3 DOL	4 years	1	Normal	None	None
Diez‐Fernandez et al. [4]	None	3 DOL	5 years	1	LD Speech delay	Not reported	Not reported
Diez‐Fernandez et al. [4]	None	2 DOL	9 months	1	Normal	None	None
Baertling et al. [5]	None	18 months	18 months	1	Patient passed away after the first MDE. Hyperammonemia and metabolic acidosis resolved in less than 24 h after the initial presentation. The encephalopathy and hypertension worsened even after the correction of the metabolic defects. He developed seizures on 3 DOA. Increased intracranial pressure was detected. Repeat MRI revealed severe brain herniation.	N/A	N/A
Konanki et al. [6]	None	3 DOL	18 months	1	Normal growth and development	No protein restriction (protein intake 2.5 g/kg/day)	Not reported
Konanki et al. [6]	Multiple	22 months	22 months	Multiple	Patient passed away during last MDE	N/A	N/A
Konanki et al. [6]^c^	None	2 DOL	4 months	1	Normal growth and development	On breast feeding. No protein restriction	Sodium benzoate
Olgac et al. [7]	None	2 DOL	18 months	1	Normal growth and development	Started on protein restriction after her first MDE. Later on, protein intake was liberalized outside MDEs. Sick‐day plan with protein restriction during illness	Carglumic acid during illnesses
Marwaha et al. [8]	None	3 DOL	9 years	1	Normal growth and development Biochemical profile normalized outside MDE Brain MRI: laminar subdural hematomas in the parietooccipital regions, posterior fossa, and posterior falx. Nonspecific hyperintensities in the deep WM in frontal, parietal and occipital lobes	Regular mixed diet Sick‐day plan	Discharged home on carnitine supplements (discontinued at the age of 3 months). Riboflavin (100–150 mg/day, discontinued at 3.6 years) No long‐term medications since the age of 3.6 years
Marwaha et al. [8]^d^	None	2 DOL	19 months	1	Normal growth and development Biochemical profile normalized outside MDE Brain MRI 4 DOL: normal	Regular mixed diet Sick‐day plan	None
Miller et al. [10]	None	4 DOL	5 years	1	Normal growth and development	Regular mixed diet Sick‐day plan	None
Miller et al. [10]	None	19 months	3 years	1	Normal growth and development Biochemical profile normalized outside MDE	Regular mixed diet Sick‐day plan	None
Sredkova et al. [11] (Poster Presentation ICIEM 2021)	None	3‐years‐7‐months	3‐years‐7‐months	1	Normal growth and development Normal biochemical investigations during febrile illnesses	Low protein diet Sick‐day management (high calorie, lipid‐rich formula)	l‐carnitine
Singanamalla et al. [12]^e^	None	3 DOL	18 months	1	Normal growth and development Normal neurological examination Continued to have elevated ammonia and lactate outside MDEs	Not reported	Sodium benzoate Carnitine Riboflavin Zinc
Sequeira [13]	None	2 DOL	4‐years‐6‐months	1	Normal growth and development	Protein restriction for 6 months after the initial MDE, followed by regular diet.	Sodium benzoate for 6 months after the initial MDE, followed by regular diet.
Stockdale et al. [14]^f^	6	5‐years‐9‐months	5‐years‐9‐months	7	Not reported. Brain MRI (MDE2): bilateral cerebral cortical cytotoxic edema	Protein restriction (0.9–1 g/kg/day) Sick‐day plan	Glycerol phenylbutyrate Sodium benzoate
Ibrahim et al. [9]	None	N/A	11 months	None	Normal growth and development	Regular infant formula	Not reported
Semenova et al. [15]	None	3 DOL	5 years	1	Normal growth and development Normal ammonia, PAA and UOA EEG: multi‐regional epileptiform discharges	Not reported	None
Urmila et al. [16]^g^	1	3 DOL	14 months	1	At 7 months: abnormal movements, EEG suggestive of bilateral generalized epileptiform activity. At 8 months: infantile spasm & developmental delay Microcephaly, hypotonia Brain MRI: mild cortical atrophy EEG: modified hypsarrhythmia during sleep At 9 months: developmental delay, normal ammonia and lactate	Sick‐day plan (IV dextrose, protein‐free diet, avoidance of fasting)	AEMs
Ganesh and Nrayanan [17]	None	3 months	18 months	1	Normal development Normal biochemical profile Normal brain MRI	Not reported	Sodium benzoate Carnitine
Al‐Thihli et al. [18]^h^	Recurrent hyperammonemia	Not reported	5 years	Multiple	GDD Microcephaly FTT Normal ammonia outside MDE	Not reported	Not reported
Al‐Thihli et al. [18]^h^	Recurrent hyperammonemia	Not reported	5 years	Multiple	Severe GDD Microcephaly FTT Mild ammonia and lactate elevation outside MDE	Not reported	Not reported
Al‐Thihli et al. [18]^h^	Recurrent hyperammonemia	Not reported	3 years	Multiple	GDD FTT Normal HC Normal ammonia outside MDE	Not reported	Not reported
Al‐Thihli et al. [18]	None	2 DOL	1 year	1	Normal neurodevelopment Normal HC FTT Normal ammonia outside MDE	Not reported	Not reported
Al‐Thihli et al. [18]^h^	Recurrent hyperammonemia	Not reported	4 years	Multiple	Mild LD Normal growth Normal HC Normal ammonia outside MDE	Not reported	Not reported
Al‐Thihli et al. [18]^h^	Recurrent hyperammonemia	Not reported	3 years	Multiple	Normal neurodevelopment Normal growth Normal HC Normal ammonia outside MDE	Not reported	Not reported
Al‐Thihli et al. [18]^h^	Recurrent hyperammonemia	2 DOL	7 years	Multiple	Normal neurodevelopment Normal HC FTT Normal ammonia outside MDE	Not reported	Not reported
Al‐Thihli et al. [18]	None	3 DOL	3 years	1	Normal neurodevelopment Normal growth Normal HC Normal ammonia outside MDE	Not reported	Not reported
Al‐Thihli et al. [18]	None	5 years	5 years	1	GDD Microcephaly FTT Normal ammonia outside MDE	Not reported	Not reported
Al‐Thihli et al. [18]	None	2 DOL	8 years	1	Normal neurodevelopment Normal growth Normal HC Normal ammonia outside MDE	Not reported	Not reported
Al‐Thihli et al. [18]	None	3 DOL	4 years	1	Normal neurodevelopment Normal growth Normal HC Normal ammonia outside MDE	Not reported	Not reported
Al‐Thihli et al. [18]	None	3 DOL	14 years	1	Normal neurodevelopment Normal growth Normal HC Normal ammonia outside MDE	Not reported	Not reported
Al‐Thihli et al. [18]	None	3 DOL	17 years	1	Normal neurodevelopment Normal HC FTT Normal ammonia outside MDE	Not reported	Not reported
Al‐Thihli et al. [18]	None	3 DOL	13 years	1	Normal neurodevelopment Normal growth Normal HC Normal ammonia outside MDE	Not reported	Not reported
Al‐Thihli et al. [18]^i^	N/A	N/A	27 years	N/A	Normal neurodevelopment Normal growth Normal HC Normal ammonia outside MDE	N/A	N/A
Al‐Thihli et al. [18]^i^	N/A	N/A	30 years	N/A	Normal neurodevelopment Normal growth Normal HC Normal ammonia outside MDE	N/A	N/A
Al‐Thihli et al. [18]	None	4 years	4 years	1	GDD Failure to thrive Normal HC Normal ammonia outside MDE	Not reported	Not reported
Al‐Thihli et al. [18]^h^	Recurrent hyperammonemia	Not reported	14 years	Multiple	GDD Normal growth Normal HC Normal ammonia outside MDE	Not reported	Not reported
Mathew et al. [19]	None	3 DOL	40 DOL	1	Ongoing neurological abnormalities: central and peripheral hypotonia, poor suck. Brain MRI was not repeated after initial MDE	Breast feeding	AEM: Levetiracetam and phenytoin
Phadke et al. [20]	None	4 DOL	5 years	1	Normal growth and development	None	None
Majety et al. [21]	None	2 DOL	2 DOL	1	Patient deceased	N/A	N/A
Abdulwahhab et al. [22]	None	3 DOL	5 weeks	1	Normal growth and development	Exclusive breast feeding. No protein restriction	Carglumic acid Biotin Riboflavin CoQ10
Keehan et al. [23]	None	18 h	4 months	1	Normal development and growth Repeat PAA & UOA normalized	None	None
Manoy et al. [24]	None	N/A	4 years	1	Normal growth and development	Initially managed via protein restriction and carnitine supplementation (discontinued).	None
Manoy et al. [24]	None	N/A	3 years	None	Normal growth and development	Sick‐day protocol during illness.	None
Manoy et al. [24]	None	4 DOL	14 months	None	Normal growth and development	Sick‐day protocol during illness.	None
Bin Hadyan et al. [25]	None	N/A	4 years	None	Axial hypotonia. GDD Normal growth Brain MRI: Marked thinning of corpus callosum. Mild periventricular leukomalacia	N/A	N/A
Bin Hadyan et al. [25]	None	N/A	7 years	None	Microcephaly (at 3rd centile). Continued to have significant speech delay, mild to moderate intellectual disability Motor skills improved Brain MRI: minor abnormalities	N/A	N/A
Bin Hadyan et al. [25]	None	N/A	4 years	None	Mild speech delay Normal growth and physical examination	N/A	N/A
Fragoso et al. [26]	None	2 DOL	3 weeks	1	Normal neurodevelopmental outcome Brain MRI (at 3 weeks): complete resolution of abnormalities found on initial MDE	Not reported	Not reported
Fragoso et al. [26]	None	3 DOL	40 months	1	Normal neurodevelopmental outcome	Not reported	Not reported
Fragoso et al. [26]	1	1 year	16 years	2	LD Microcephaly	Not reported	Not reported
Fragoso et al. [26]	None	13 months	24 months	1	Normal neurodevelopmental outcome Brain MRI (11 months after initial MDE): mild residual gliotic changes in bilateral centrum semiovale and corona radiata, resolution of previously noted abnormalities	Not reported	Not reported
Fragoso et al. [26]	None	1 DOL	7 years	1	Severe GDD Axial hypotonia Central hypertonia Microcephaly Stereotypic behavior and autistic features	Not reported	Not reported
Fragoso et al. [26]	None	4 DOL	14 months	1	GDD Microcephaly Brain MRI (14 months after initial MDE): extensive residual gliotic changes in the bilateral periorlandic and insular cortices, subcortical WM, putamina, globi pallidi, and superior peduncles, new involvement of bilateral caudet heads and left thalamus	Not reported	Not reported
Fragoso et al. [26]	1	6 years	7 years	2	Normal neurodevelopmental outcome	Not reported	Not reported
Fragoso et al. [26]	None	6 DOL	6 months	1	Normal neurodevelopmental outcome	Not reported	Not reported
Fragoso et al. [26]^j^	None	3 DOL	24 months	1	GDD Hypotonia Recurrent symptomatic hypoglycemia	Not reported	Not reported

Abbreviations: DOA, day of admission; DOL, day of life; FTT, failure to thrive; GDD, global developmental delay; HC, head circumference; LD, learning difficulties; MDEs, metabolic decompensation episodes; WM, white matter.

^a^ Patient continued to have infrequent episodes of vomiting and ketoacidosis without hyperammonemia or elevated lactate.

^b^ Second MDE were milder than the initial MDE.

^c^ Patient was found to have elevated ammonia at 174 μmol/L on routine assessment at 1 month of age. No clinical symptoms were reported.

^d^ Patient also reported in Ibrahim et al. [9] at 2 years of age.

^e^ Patient was found to have elevated ammonia up to 340 μmol/L in routine assessment.

^f^ In 2 episodes, the patient required PICU admission and was on hemofiltration for ammonia detoxification. In episode 2, he had increased intracranial pressure and cerebral edema.

^g^ Patient presented with symptoms not usually seen in MDEs. No further details were provided in the report.

^h^ Patients with recurrent hyperammonemia were not specified in the article.

^i^ Asymptomatic adults diagnosed through cascade family testing.

^j^ Patient has a concurrent diagnosis of MCAD deficiency.

### 4.2 Clinical Presentation

The hallmark of CA‐VA deficiency is an acute MDE characterized by hyperammonemic encephalopathy (86.5%), hyperlactatemia (79.7%), elevated ketones (49.3%), and mixed acid–base disturbances (Table 2) [3, 4, 13, 18, 20, 28]. Hypoglycemia was inconsistent across reported cases, occurring in only 37.7% of cases, and was not correlated with the severity of MDEs [3, 4, 5, 6, 7, 8, 10, 13, 15, 16, 18, 20, 22, 23]. Atypical presentations included hypoammonemia (*n* = 1) [21], normal lactate (*n* = 8) [18, 25], hyperglycemia (*n* = 1) [11], and cases without MDEs (*n* = 8) [25]. Case 1 exhibited recurrent hyperCKemia during MDEs, a finding observed in ten other cases, suggesting a possible underrecognized feature of CA‐VA deficiency [18, 23]. However, concurrent myositis in the context of febrile illnesses cannot be excluded. Therefore, routine assessment of CK and urine myoglobin levels in patients with CA‐VA deficiency is warranted. Liver transaminases were normal in our cases, consistent with most published reports (*n* = 8/13) [3, 11, 14, 27, 28]. Transaminitis was reported in five cases, one of which had accompanying hepatomegaly [6, 15, 17, 19, 24].

### 4.3 Biochemical Profile

PAA showed elevated glutamine (59.4%) with low‐normal citrulline, consistent with secondary CPS1 deficiency, although arginine and ornithine levels varied among patients (Table 2) [4, 7, 10, 13]. The elevation in alanine (53.1%) and proline (28.1%) is consistent with the lactate level, reflecting mitochondrial dysfunction [3, 4, 6, 8, 13, 15, 22, 23, 28]. Analysis of UOA typically reveals marked ketonuria (84.4%) and lactic aciduria (81.3%), and in some cases, metabolites suggestive of multiple carboxylase (31%) or pyruvate carboxylase deficiency (34.4%) may be present [3, 28], while in other cases, the UOA profile remained normal (*n* = 9) [16, 18]. Urine orotic acid was either normal (*n* = 21) [3, 4, 8, 13, 14, 19, 26], similar to our cases, or mildly elevated (*n* = 1) [8], suggesting reduced carbamoyl phosphate production in CA‐VA deficiency, as opposed to primary urea cycle defects. Newborn screening could theoretically identify individuals with CA‐VA deficiency through elevated C3 and C5‐OH levels, as seen in multiple carboxylase defects [3, 8, 28]. However, both newborn screening and serial acylcarnitine levels were normal in our cases, even during acute MDEs (case 1 MDE2: C3:0.67 μmol/L (0.56–3.22), C5‐OH: 0.17 μmol/L (0.07–0.56), case 2 MDE1: C3:0.41 μmol/L (0.06–0.94), C5‐OH:0.06 μmol/L (0.02–0.08)), consistent with all previously reported cases (*n* = 11) [10, 13, 18, 22, 23, 24]. Moreover, 58% of cases present between the second and fourth days of life, often before newborn screening results are available [8, 28].

### 4.4 Neurological Manifestations and Neuroimaging

Seizures and epileptic encephalopathy have been documented in cases with CA‐VA deficiency during MDE (*n* = 9/67), mostly provoked by hyperammonemic crises (Table 2) [6, 15, 16, 17, 26]. Although some cases demonstrate complete recovery (*n* = 3) [15, 17], chronic epilepsy (*n* = 2) [16, 18] and fatal refractory seizures (*n* = 1) [5] have been reported. EEG data remain among the most sparsely reported variables in the literature, documented only in four cases. Diffuse cerebral dysfunction was the predominant EEG finding in 2/4 cases, of which one case had electrographic seizures without overt clinical manifestations, raising the importance of EEG monitoring during MDEs [6, 15]. Although both clinical and electrical abnormalities are expected to improve with ammonia normalization, persistent multiregional epileptiform discharges have been reported (*n* = 1), indicating post‐MDE cortical hyperexcitability related to prolonged hyperammonemic injury [15]. Hypsarrhythmia consistent with West syndrome (*n* = 1) has also been documented [16]. In contrast, the EEG obtained during the initial MDE in case 1 was unremarkable.

Normal neuroimaging was observed in six cases, of which three had single neonatal‐onset MDE (brain MRI, *n* = 3) [8, 12, 17] and three had recurrent neonatal‐onset MDE (computed tomography [CT], *n* = 3, Table 2) [18]. All cases with normal neuroimaging had normal neurological outcomes, except for one case with mild learning difficulties. Abnormal brain MRI was evident in 21 cases and fell along a severity spectrum. On the milder end, some patients displayed images consistent with hyperammonemic encephalopathy with selective involvement of the cerebral cortex and the subcortical white matter, reflecting a recoverable injury (*n* = 9) [14, 18, 26]. More severe cases demonstrated T2 signal hyperintensities and diffusion restriction extending to the thalami, basal ganglia, and brainstem, consistent with acute infarcts and indicating more extensive, potentially irreversible damage (*n* = 8) [6, 18, 26]. Baertling et al. [5] fatal case represents the most severe end of the spectrum, with neuroimaging showing severe brain herniation with increased intracranial pressure, indicating the progression of brain edema despite the normalization of ammonia and lactate levels. Fragoso et al. [26] formalized this spectrum into two radiological patterns, with the favorable outcome group (FOG) defined by selective involvement of the cortical and subcortical regions of the insular/peri‐insular, Sylvian/peri‐Sylvian, and peri‐Rolandic cortex. In contrast, the unfavorable outcome group (UOG) was characterized by more extensive lesions in these areas, as well as deep gray matter and brainstem involvement. These changes may result from a combination of factors, including hyperammonemia, hypoglycemia, carboxylase deficiency, and organic acid accumulation, causing cellular energy failure, reduced Na+/K+ ATPase activity, and ultimately cytotoxic cell edema [29]. While some cases demonstrated complete radiological resolution [26], chronic brain MRI changes have been reported, including cortical atrophy (*n* = 1) [16] and thinning of the corpus callosum and periventricular leukomalacia (*n* = 1; Table 3) [25]. Brain MRI in case 1 remained unremarkable throughout MDEs. In case 2, the initial brain MRI revealed nonspecific white matter injury that fully resolved on repeat imaging at 11 years of age, consistent with the FOG.

Brain MRS has been reported in 6 cases, with normal results in one, similar to our two cases (Table 2) [26]. Elevated lactate (*n* = 1) [5], elevated glutamine and lactate (*n* = 1) [10], elevated glutamine/glutamate (*n* = 2) [26], and choline peak (*n* = 1) [19] have been reported, reflecting anaerobic metabolism defect, excitotoxicity and increased membrane turnover [30].

### 4.5 Metabolic Decompensation Episodes (MDEs)

The majority of cases (44/67) experienced a single MDE, after which they remained metabolically stable with a normal biochemical profile, even during subsequent febrile illnesses (Table 3) [4, 6, 8, 10, 11, 12, 13, 15, 20, 22, 23, 27]. Clinical stability in these cases may be attributed to the progressive compensatory upregulation of CA‐VB activity [2]. A further six cases experienced a total of two MDEs before achieving similar metabolic stability [3, 4, 26]. At the severe end of the spectrum, 13.3% of cases had recurrent MDEs triggered by catabolic stress, most notably the seven cases reported by Al‐Thihli et al. [18] and the case reported by Stockdale et al. [14], who experienced seven episodes, the highest documented MDE count. Three fatalities were reported despite proper management, all occurring before the age of 2 years: in the neonatal period (*n* = 1) [21], at 18 months (*n* = 1) [5], and at 22 months (*n* = 1) [6].

Although most cases showed a decrease in the severity of MDEs with age [3, 4], one case continued to experience severe MDEs requiring admission to PICU [14]. The age at the last MDE varied across the reported cases. Most cases had their last MDE during the neonatal (*n* = 28/67) and infantile (*n* = 8/67) periods [3, 4, 6, 7, 8, 10, 12, 13, 15, 16, 17, 19, 20, 22, 23, 24, 26]. However, MDEs have been reported as late as 4 years 1 month, 5 years 9 months, and 6 years, confirming that the recurrence risk extends into childhood despite periods of clinical stability [4, 14, 26].

Case 2 had a single neonatal MDE and remained metabolically stable thereafter, consistent with most reported cases. In contrast, case 1 experienced four MDEs, the last occurring at the age of 8 years, extending the upper age limit for MDE recurrence beyond any previously reported case. This phenotypic discordance between two siblings sharing an identical biallelic variant in *CA5A* gene is not novel. Al‐Thihli et al. [18] documented substantial intrafamilial variability among 18 cases with the same founder mutation c.59G>A (Table 3). Interfamilial variability is equally striking; for instance, cases with the variant c.721G>A have been reported across different ethnic backgrounds and displayed markedly different clinical courses, with no consistent genotype–phenotype correlation. Several hypotheses may explain the phenotypic heterogeneity between cases 1 and 2. Gene modifiers acting independently of *CA5A* gene may influence the threshold for metabolic decompensation [18]. Additionally, environmental factors, including the frequency, severity, and pathogen burden of intercurrent illnesses in childhood, may determine how often the catabolic threshold is breached. Sex‐related differences in hepatic enzyme expression may influence the developmental upregulation of CA‐VB in the context of CA‐VA deficiency, leading to a higher compensatory plateau in males and earlier protection against MDE [31, 32, 33].

### 4.6 Impact on Neurodevelopment and Growth

Normal developmental outcomes were reported in 67.2% (*n* = 45/67) of cases, the majority of whom (*n* = 33/45) experienced no further MDEs beyond their initial presentation (Table 3) [3, 4, 6, 7, 8, 10, 11, 12, 13, 15, 17, 18, 20, 22, 23, 24]. The remaining cases with normal outcomes had two or more MDEs (*n* = 7) [4, 18]. Approximately 25% (*n* = 18) of the cases exhibited a spectrum of neurodevelopmental abnormalities. Mild intellectual disability (ID, *n* = 4) and mild to moderate global developmental delay (GDD, *n* = 6) were the most frequently reported adverse outcomes among symptomatic cases [3, 4, 18, 25]. Notably, three cases presented with isolated speech delay or speech delay with ID in the absence of MDEs, representing a distinct phenotypic subgroup with MDE‐independent neurological injury [25]. Severe GDD was documented in six cases, several of whom exhibited abnormal neuroimaging and neurological abnormalities, suggesting a more devastating outcome [18, 26]. The neurodevelopmental outcome did not correlate with the genotype, number of MDEs, age of onset, age at last MDE, duration, or severity of hyperammonemia, consistent with significant intra‐ and interfamilial variability as described earlier [16, 18, 26]. The impact on the outcome may be attributed, in part, to differences in the severity of individual MDE‐related brain injuries [18, 26].

The impact of CA‐VA deficiency on somatic growth and head circumference is underappreciated (Table 3). Microcephaly was documented in eight cases [16, 18, 25, 26]. The occurrence of severe microcephaly in the context of a single neonatal MDE raises the possibility of brain injury triggered by the acute hyperammonemia superimposed on underlying chronic bicarbonate deficiency affecting cerebral growth [18, 26]. However, microcephaly was documented in the absence of MDEs, suggesting mechanisms beyond hyperammonemia alone [25]. Although Al‐Thihli et al. [18] raised the possibility of an unidentified co‐inherited disorder as a possible etiology, the initial WES/WGS and WES reanalysis failed to identify additional clinically relevant variants in these cases, whereas comprehensive genetic testing was not performed in the remaining cases [16, 18, 25].

Normal somatic growth was documented in most cases (*n* = 45/67) [3, 4, 18], whereas failure to thrive (FTT) was encountered in eight cases [18]. Although the impact of recurrent MDE may collectively impair weight gain and linear growth, it is not sufficient to explain FTT in cases with a single MDE. Notably, most cases with FTT also have significant neurological sequelae, which may impact feeding and later contribute to calorie deficits. Furthermore, chronic impairment of pyruvate carboxylase and gluconeogenesis may affect the availability of substrates for anabolic processes. In contrast, excessive growth has been reported in one case of obesity, which may reflect the impact of the high‐fat sick‐day formula used during intercurrent illnesses [6].

Case 1 demonstrated early fatigue‐related performance variability, along with reduced upper‐ and lower‐limb strength, suggesting decreased muscle energy production. Similarly, Case 2 showed signs of increasing fatigability and distractibility during neurodevelopmental assessment in early childhood, raising the possibility of a mild attention deficit. Both cases demonstrated normal growth, intellectual function, and neurological outcomes, with improvements over time in physical and cognitive abilities, consistent with the generally favorable prognosis seen in well‐managed cases. Additionally, our cases continued to have normal ophthalmology, cardiac, hepatic, and renal assessments, consistent with most reported cases. This finding distinguishes CA‐VA deficiency from primary mitochondrial disorders, in which multi‐organ involvement is typical [34].

### 4.7 Acute and Long‐Term Management

Owing to the rare and novel nature of CA‐VA deficiency, no clinical management guidelines have yet been published. However, there is a broad consensus on the approach to acute MDEs (Table 2), which focuses on the reversal of catabolism and anabolism induction by providing high caloric intake using IV dextrose (10% at 150% maintenance) and age‐appropriate doses of IV lipids (1–2 g/kg/day), avoidance of fasting to suppress endogenous protein catabolism, and temporary protein restriction for 24–48 h with gradual introduction of essential amino acids as ammonia levels normalize [3, 28, 35]. Bicarbonate supplementation has been used as a symptomatic treatment (*n* = 9) to correct the metabolic acidosis accompanying MDEs, often prior to diagnostic confirmation [6, 7, 8, 15, 17, 21, 24]. Carglumic acid (100 mg/kg/day) provides the most targeted acute intervention [3, 4, 18, 24]. When used (*n* = 27), it significantly shortened the duration of hyperammonemia compared to standard measures alone [8, 14]. Further, it was employed proactively in three cases identified antenatally [9, 24]. While a single dose of carglumic acid has been successfully used in some cases (*n* = 2), a 3‐ to 5‐day course may be required when hyperammonemia persists, in combination with adjunct therapies and close ammonia monitoring [3, 14]. Ammonia scavengers (sodium benzoate and sodium phenylacetate), sometimes combined with l‐arginine, were used as adjunct therapies, with or without extracorporeal ammonia removal, in cases with severe, refractory hyperammonemia with progressive encephalopathy (*n* = 32) [3, 4, 18]. Mitochondrial cofactor supplementation was administered during acute MDEs in 13 cases, with variable agent selection, including l‐carnitine (*n* = 13), biotin (*n* = 6), CoQ 10 (*n* = 3), riboflavin (*n* = 3), vitamin C (*n* = 2), cobalamin (*n* = 2), thiamine (*n* = 1), folic acid (*n* = 1), and zinc (*n* = 1), reflecting the original diagnostic uncertainty given the overlap with primary mitochondrial disorders. In the absence of biochemical evidence of primary mitochondrial dysfunction, there is no established rationale to continue these agents once the diagnosis of CA‐VA deficiency is confirmed and metabolic stability is achieved.

Long‐term pharmacological intervention is not routinely required in most patients outside acute MDEs (Table 3) [3, 4, 6, 7, 8, 10, 11, 13, 19, 24]. Long‐term ammonia scavengers (*n* = 6) and protein restriction (*n* = 5) were used in a minority of cases, some of which had baseline persistent hyperammonemia or recurrent MDEs [3, 6, 12, 14, 17, 20]. Outside MDEs, long‐term administration of carglumic acid was not recommended and was used exclusively during intercurrent illnesses (*n* = 2), reflecting the episodic nature of hyperammonaemia in CA‐VA deficiency [3, 7].

All patients should be provided with a personalized sick‐day protocol during periods of febrile illness and catabolic states, consisting of high‐calorie intake from fats and carbohydrates, temporary protein restriction, strict avoidance of fasting, and clear criteria for hospital admission [28]. Although long‐term outcomes are generally favorable in well‐managed cases, ongoing surveillance is recommended given the risk of MDE recurrence into later childhood and potential neurodevelopmental sequelae.

## 5 Conclusion

CA‐VA deficiency is a rare yet clinically significant inherited metabolic disorder that presents with hyperammonemic encephalopathy, lactic acidosis, and ketosis, mostly in neonates and infants, while some patients may present later in life or remain asymptomatic until adulthood. There is noticeable clinical and biochemical heterogeneity, intrafamilial and interfamilial variability, and no clear genotype–phenotype correlation. Outcomes vary among affected individuals. While the prognosis is mostly favorable in promptly treated patients, severe and fatal outcomes have been reported. The lack of consistent biochemical or radiological signatures underscores the importance of molecular testing for the early diagnosis of CA‐VA deficiency. Consensus guidelines for the treatment and long‐term monitoring of CA‐VA deficiency are essential.

## Author Contributions

S.S. contributed to the design and concept, data collection, data analysis and interpretation, literature review, manuscript drafting, revision and editing, figures and tables preparation, and manuscript final approval and submission. A.M. contributed to the manuscript design, data analysis and interpretation, literature review, manuscript drafting, revision and editing, and supplemental data preparation. G.H. and S.S.‐I. provided clinical care and contributed to the manuscript review and final approval. C.K. provided clinical care and contributed to the design, concept, data analysis, and interpretation. R.S.‐Z. provided ongoing clinical care and contributed to the design and concept, data collection, data analysis and interpretation, manuscript revision and editing, and final review of the manuscript for submission.

## Funding

The authors have nothing to report.

## Ethics Statement

All procedures and investigations in this article were part of the routine standard of care and did not require ethical approval.

## Consent

Written informed consent and assent were obtained from all participants for inclusion in the study.

## Conflicts of Interest

The authors declare no conflicts of interest.

## Data Availability

The data that support the findings of this study are available from the corresponding author upon reasonable request.

## References

[1] W. S. Sly and P. Y. Hu , “Human Carbonic Anhydrases and Carbonic Anhydrase Deficiencies,” Annual Review of Biochemistry 64 (1995): 375–401. 10.1146/annurev.bi.64.070195.002111 7574487

[2] G. N. Shah , D. Hewett‐Emmett , J. H. Grubb , et al., “Mitochondrial Carbonic Anhydrase CA VB: Differences in Tissue Distribution and Pattern of Evolution From Those of CA VA Suggest Distinct Physiological Roles,” Proceedings of the National Academy of Sciences of the United States of America 97, no. 4 (2000): 1677–1682. 10677517 10.1073/pnas.97.4.1677 PMC26495

[3] C. D. van Karnebeek , W. S. Sly , C. J. Ross , et al., “Mitochondrial Carbonic Anhydrase VA Deficiency Resulting From CA5A Alterations Presents With Hyperammonemia in Early Childhood,” American Journal of Human Genetics 94, no. 3 (2014): 453–461. 24530203 10.1016/j.ajhg.2014.01.006 PMC3951944

[4] C. Diez‐Fernandez , V. Rüfenacht , S. Santra , et al., “Defective Hepatic Bicarbonate Production due to Carbonic Anhydrase VA Deficiency Leads to Early‐Onset Life‐Threatening Metabolic Crisis,” Genetics in Medicine 18, no. 10 (2016): 991–1000. 26913920 10.1038/gim.2015.201

[5] F. Baertling , M. Wagner , T. Brunet , et al., “Fatal Metabolic Decompensation in Carbonic Anhydrase VA Deficiency Despite Early Treatment and Control of Hyperammonemia,” Genetics in Medicine 22, no. 3 (2020): 654–655. 31641285 10.1038/s41436-019-0677-9

[6] R. Konanki , R. R. D. Akella , N. Panigrahy , D. K. Chirla , S. Mohanlal , and R. Lankala , “Mitochondrial Carbonic Anhydrase VA Deficiency in Three Indian Infants Manifesting Early Metabolic Crisis,” Brain & Development 42, no. 7 (2020): 534–538. 32381389 10.1016/j.braindev.2020.04.007

[7] A. Olgac , C. S. Kasapkara , M. Kilic , et al., “Carbonic Anhydrase VA Deficiency: A Very Rare Case of Hyperammonemic Encephalopathy,” Journal of Pediatric Endocrinology & Metabolism 33, no. 10 (2020): 1349–1352. 32809955 10.1515/jpem-2020-0117

[8] A. Marwaha , J. Ibrahim , T. Rice , et al., “Two Cases of Carbonic Anhydrase VA Deficiency—An Ultrarare Metabolic Decompensation Syndrome Presenting With Hyperammonemia, Lactic Acidosis, Ketonuria, and Good Clinical Outcome,” JIMD Reports 57, no. 1 (2021): 9–14. 33473334 10.1002/jmd2.12171 PMC7802620

[9] J. Ibrahim , S. Ratko , N. Karp , et al., “Carbonic Anhydrase (CA‐VA) Deficiency: An Under Recognised Cause of Neonatal Hyperammonemia With Excellent Outcome on Proactive Management,” Archives of Disease in Childhood 107 (2022): 200–201.

[10] M. J. Miller , S. M. Luu , and B. H. Graham , “Acute Hyperammonemia, Lactic Acidosis, and Ketoacidosis in a Developmentally Normal Child,” Clinical Chemistry 67, no. 11 (2021): 1572–1574. 34726698 10.1093/clinchem/hvab112

[11] T. D. Maria Sredkova , M. Ivanova , and D. Avdjieva‐Tzavella , “A Case Report of Carbonic Anhydrase VA Deficiency,” Journal of Inherited Metabolic Disease 44, no. S1 (2021): 147.

[12] B. Singanamalla , A. G. Saini , S. V. Attri , R. Suthar , and K. Mukhopadhyay , “Carbonic Anhydrase‐VA Deficiency: A Close Mimicker of Urea Cycle Disorders,” Annals of Indian Academy of Neurology 24, no. 5 (2021): 820–821. 35002167 10.4103/aian.AIAN_563_20 PMC8680920

[13] S. Sequeira , “Mitochondrial Carbonic Anhydrase VA Deficiency in Neonatal Hyperammonemic Encephalopathy: Case Report,” Portuguese Journal of Pediatrics 52 (2021): 117–121.

[14] C. Stockdale , A. Bowron , M. Appleton , R. Richardson , and M. Anderson , “Recurrent Hyperammonemia in a Patient With Carbonic Anhydrase VA Deficiency,” Journal of Inherited Metabolic Disorders Report 63, no. 6 (2022): 536–539. 10.1002/jmd2.12322 PMC9626664 36341166

[15] N. Semenova , A. Marakhonov , M. Ampleeva , et al., “Hyperammonemia in Russia due to Carbonic Anhydrase VA Deficiency Caused by Homozygous Mutation p.Lys185Lys (c.555G>A) of the CA5A Gene,” International Journal of Molecular Sciences 23, no. 23 (2022): 15026. 36499355 10.3390/ijms232315026 PMC9739189

[16] N. Mani Urmila , D. Kewalramani , U. Balakrishnan , and R. K. Manokaran , “A Case of Carbonic Anhydrase Type VA Deficiency Presenting as West Syndrome in an Infant With a Novel Mutation in the CA‐VA Gene,” Epilepsy & Behavior Reports 20 (2022): 100573. 36411877 10.1016/j.ebr.2022.100573 PMC9674490

[17] R. Ganesh and R. Karthik Narayanan , “An Extremely Rare Cause of Hyperammonemic Encephalopathy in an Infant,” Indian Journal of Pediatrics 91, no. 1 (2024): 88. 37610685 10.1007/s12098-023-04818-z

[18] K. Al‐Thihli , N. Al Hashmi , A. Al Balushi , et al., “A Founder Mutation in CA5A Causing Intrafamilial and Interfamilial Phenotypic Variability in a Cohort of 18 Patients With Carbonic Anhydrase VA Deficiency,” JIMD Reports 65, no. 4 (2024): 226–232. 38974611 10.1002/jmd2.12426 PMC11224491

[19] R. P. Mathew , P. Ranya Raghavendra , B. Disha , A. Dalal , and P. Govindaraj , “A Unique Case of Hyperammonemia due to CA5A Deficiency: Impact of Coexisting Gene Mutations, Pseudogene, and Microdeletion,” American Journal of Medical Genetics. Part A 194, no. 11 (2024): e63809. 38949089 10.1002/ajmg.a.63809

[20] A. Phadke , A. Kumble , A. Varghese , M. Jacob , and A. Siriac , “Rare Yet Reversible: CA5A Deficiency Presenting as Neonatal Metabolic Crisis,” Indian Journal of Child Health 12, no. 9 (2025): 118–120.

[21] M. Majety , L. C. Nagalla , S. Ravipati , K. P. K. N. Venkataramana , and S. Suryanarayana , “Atypical/Mysterious Persentation of Hypoammonemia in CAVA‐Defcient Neonate: A Case Report,” Journal of Pediatrics and Pediatric Medicine 5, no. 4 (2025): 1–4.

[22] S. Baheer Abdulwahhab , A. Ahmed , and T. Ben Omran , “Neonatal Presentation of a Case of Carbonic Anhydrase VA Deficiency,” Cureus 17, no. 8 (2025): e90845. 40862046 10.7759/cureus.90845 PMC12375167

[23] L. Keehan , E. Null , L. Chilakamarri , et al., “Carbonic Anhydrase VA Deficiency due to a Novel CA5A Variant,” Molecular Genetics and Metabolism Reports 45 (2025): 101259. 41113665 10.1016/j.ymgmr.2025.101259 PMC12528933

[24] S. Manoy , T. Minto , K. Demetriou , et al., “Antenatal and Neonatal Management of Siblings With Carbonic Anhydrase VA Deficiency,” JIMD Reports 67, no. 2 (2026): e70076. 41737902 10.1002/jmd2.70076 PMC12928768

[25] M. F. Bin Hadyan , M. A. Saleh , S. Aldalaqan , et al., “A Novel Homozygous CA5A Gene Deletion in Carbonic Anhydrase VA Deficiency Presenting as Developmental Delay Without Metabolic Crisis,” Molecular Genetics and Metabolism Reports 46 (2026): 101294. 41736722 10.1016/j.ymgmr.2026.101294 PMC12926607

[26] D. C. Fragoso , E. al‐Ajmi , A. M. Cardenas , et al., “Neuroimaging Findings in Carbonic Anhydrase VA Deficiency: A Case Series Highlighting Diagnostic and Prognostic Patterns in a Potentially Reversible Mitochondrial Dysfunction,” AJNR. American Journal of Neuroradiology 47, no. 1 (2026): 200–207. 41022560 10.3174/ajnr.A8948 PMC12767740

[27] D. Kalliope , A. Selvanathan , M. Lipke , et al., “Carbonic Anhydrase VA Deficiency in a Neonate With Hyperammonaemia, Lactic Acidosis and Severe Ketoacidosis,” Journal of Inherited Metabolic Disease 44, no. S1 (2021): 187.

[28] C. van Karnebeek and J. Haberle , Carbonic Anhydrase VA Deficiency, ed. M. P. Adam (GeneReviews((R)), 1993). 25834911

[29] S. Gaddamanugu , O. Shafaat , H. Sotoudeh , et al., “Clinical Applications of Diffusion‐Weighted Sequence in Brain Imaging: Beyond Stroke,” Neuroradiology 64, no. 1 (2022): 15–30. 34596716 10.1007/s00234-021-02819-3 PMC8484843

[30] L. M. Lai , A. L. Gropman , and M. T. Whitehead , “MR Neuroimaging in Pediatric Inborn Errors of Metabolism,” Diagnostics (Basel) 12, no. 4 (2022): 861. 35453911 10.3390/diagnostics12040861 PMC9027484

[31] T. L. Conforto and D. J. Waxman , “Sex‐Specific Mouse Liver Gene Expression: Genome‐Wide Analysis of Developmental Changes From Pre‐Pubertal Period to Young Adulthood,” Biology of Sex Differences 3 (2012): 9. 22475005 10.1186/2042-6410-3-9 PMC3350426

[32] D. J. Waxman and M. G. Holloway , “Sex Differences in the Expression of Hepatic Drug Metabolizing Enzymes,” Molecular Pharmacology 76, no. 2 (2009): 215–228. 19483103 10.1124/mol.109.056705 PMC2713118

[33] M. Blencowe , X. Chen , Y. Zhao , et al., “Relative Contributions of Sex Hormones, Sex Chromosomes, and Gonads to Sex Differences in Tissue Gene Regulation,” Genome Research 32, no. 5 (2022): 807–824. 35396276 10.1101/gr.275965.121 PMC9104702

[34] G. S. Gorman , P. F. Chinnery , S. DiMauro , et al., “Mitochondrial Diseases,” Nature Reviews. Disease Primers 2 (2016): 16080. 10.1038/nrdp.2016.80 27775730

[35] K. A. Kripps , P. R. Baker, II , J. A. Thomas , et al., “REVIEW: Practical Strategies to Maintain Anabolism by Intravenous Nutritional Management in Children With Inborn Metabolic Diseases,” Molecular Genetics and Metabolism 133, no. 3 (2021): 231–241. 33985889 10.1016/j.ymgme.2021.04.007

